# Structure of PLA2R reveals presentation of the dominant membranous nephropathy epitope and an immunogenic patch

**DOI:** 10.1073/pnas.2202209119

**Published:** 2022-07-11

**Authors:** Maryline Fresquet, Michael P. Lockhart-Cairns, Samuel J. Rhoden, Thomas A. Jowitt, David C. Briggs, Clair Baldock, Paul E. Brenchley, Rachel Lennon

**Affiliations:** ^a^Wellcome Centre for Cell-Matrix Research, The University of Manchester, Manchester, M13 9PT, United Kingdom;; ^b^Division of Cell-Matrix Biology and Regenerative Medicine, School of Biological Sciences, Faculty of Biology Medicine and Health, The University of Manchester, Manchester Academic Health Science Centre, Manchester, M13 9PT, United Kingdom;; ^c^Signalling and Structural Biology Lab, The Francis Crick Institute, London, NW1 1AT, United Kingdom;; ^d^Division of Cardiovascular Sciences, The University of Manchester, Manchester, M13 9PT, United Kingdom;; ^e^Department of Paediatric Nephrology, Royal Manchester Children’s Hospital, Manchester University Hospitals NHS Foundation Trust, Manchester Academic Health Science Centre, Manchester, M13 9WL, United Kingdom

**Keywords:** PLA2R epitope, antigenic residues, 3.4 Å, resolution croyEM structure

## Abstract

Membranous nephropathy is an autoimmune kidney disease caused by autoantibodies targeting antigens present on glomerular podocytes, instigating a cascade leading to glomerular injury. The most prevalent circulating autoantibodies in membranous nephropathy are against phospholipase A2 receptor (PLA2R), a cell surface receptor. The dominant epitope in PLA2R is located within the cysteine-rich domain, yet high-resolution structure-based mapping is lacking. In this study, we define the key nonredundant amino acids in the dominant epitope of PLA2R involved in autoantibody binding. We further describe two essential regions within the dominant epitope and spacer requirements for a synthetic peptide of the epitope for drug discovery. In addition, using cryo-electron microscopy, we have determined the high-resolution structure of PLA2R to 3.4 Å resolution, which shows that the dominant epitope and key residues within the cysteine-rich domain are accessible at the cell surface. In addition, the structure of PLA2R not only suggests a different orientation of domains but also implicates a unique immunogenic signature in PLA2R responsible for inducing autoantibody formation and recognition.

## Significance

• Phospholipase A2 receptor (PLA2R) is the major autoantigen responsible for causing the rare autoimmune kidney disease, membranous nephropathy (MN).
• PLA2R autoantibodies primarily bind to a dominant epitope located within a 28-amino acid peptide in the cysteine-rich domain of PLA2R.
• We generated a minimal peptide necessary for binding autoantibodies with high affinity for developing future therapies.
• We report the high-resolution structure of PLA2R with its domain orientation and define key regions within the domain epitope critical for its immunogenicity in MN.

Membranous nephropathy (MN) is a rare kidney disease in which autoantibodies are generated against glomerular antigens, and immune complexes are deposited in the glomerular basement membrane, triggering glomerular injury and dysfunction (1). MN is the most common cause of adult nephrotic syndrome in the Caucasian population (2, 3), and in 60% of patients, kidney function deteriorates, with ultimately 30–40% developing end-stage kidney disease within 10 y of the initial diagnosis (4, 5). Most instances of MN are associated with the presence of circulating autoantibodies against a podocyte cell surface receptor, phospholipase A2 receptor (PLA2R) (6, 7). The PLA2R autoantibodies primarily bind to a dominant epitope located within a 31-amino acid peptide in the cysteine-rich domain (CysR) of PLA2R (8). Autoantibodies that recognize additional domains in PLA2R located in the C-type lectin domains (CTLD) 1, 7, and 8 have been identified, although quantitative ELISA (enzyme-linked immunosorbent assay) shows the antibody response to CTLDs to be significantly lower than to the CysR epitope (9–11). Therefore, current evidence suggests that the N-terminal CysR epitope is the dominant epitope recognized by the immune system, and over time, the other epitopes become involved in a process of epitope spreading (12).

Although MN antibodies are described and used as a diagnostic tool, there remains an unmet need for more targeted therapies in MN because current treatments are limited to supportive interventions and nonspecific immunosuppression with severe side effects (13). In addition to understanding the immunogenicity of PLA2R in MN, detailed knowledge of the dominant B-cell epitope will provide a foundation for the development of new immunotherapies for specific removal or inhibition of the pathogenic autoantibody. However, there are issues with targeting the CysR epitope, which need to be overcome. The length of the peptide makes manufacturing difficult, potentially expensive, time consuming, and difficult to scale up production by solid-phase peptide synthesis (14). The presence of a disulphide bond within the peptide can also impact its stability. To address these potential issues, it is important to further define the fundamental residues for autoantibody binding within the dominant epitope sequence to shorten the peptide for therapeutic use.

PLA2R is a member of the mannose receptor family, a five-protein subgroup of the C-type lectin superfamily comprising the mannose receptor, Endo180, DEC-205, PLA2R. and FcRY (15, 16). All family members contain a homologous CysR domain, fibronectin type II (FnII) domain, multiple sequential CTLDs, and a short intracellular C-terminal tail. Mannose receptor family proteins are all endocytic receptors, with pH-dependent conformational changes thought to be important in binding to their respective targets (17–20). Each of the mannose receptor family members has different binding targets despite sharing a high level of sequence similarity (21–26).

Current structural models of PLA2R resolve the structure to ∼10 Å (27). One study solved the structure of CTLD7 by X-ray diffraction, and it was used as a tool to investigate epitope sites in that domain (28). However, a higher resolution structure of PLA2R allowing the MN autoantibody epitope to be accurately located in the CysR domain is still required.

Therefore, we sought to determine 1) the key nonredundant amino acids in the dominant epitope of PLA2R involved in autoantibody binding; 2) the presence of substructure of the primary sequence essential for antibody binding; and 3) a high-resolution cryo-electron microscopy (cryoEM) structure of the full-length extracellular domain (ECD) of PLA2R to define the domain orientations around CysR in relation to CTLD1, 7, and 8.

## Results

### Peptide microarray of the dominant MN epitope reveals two linear short sequences with key amino acid residues required for PLA2R autoantibody binding.

**Fig. 1.**
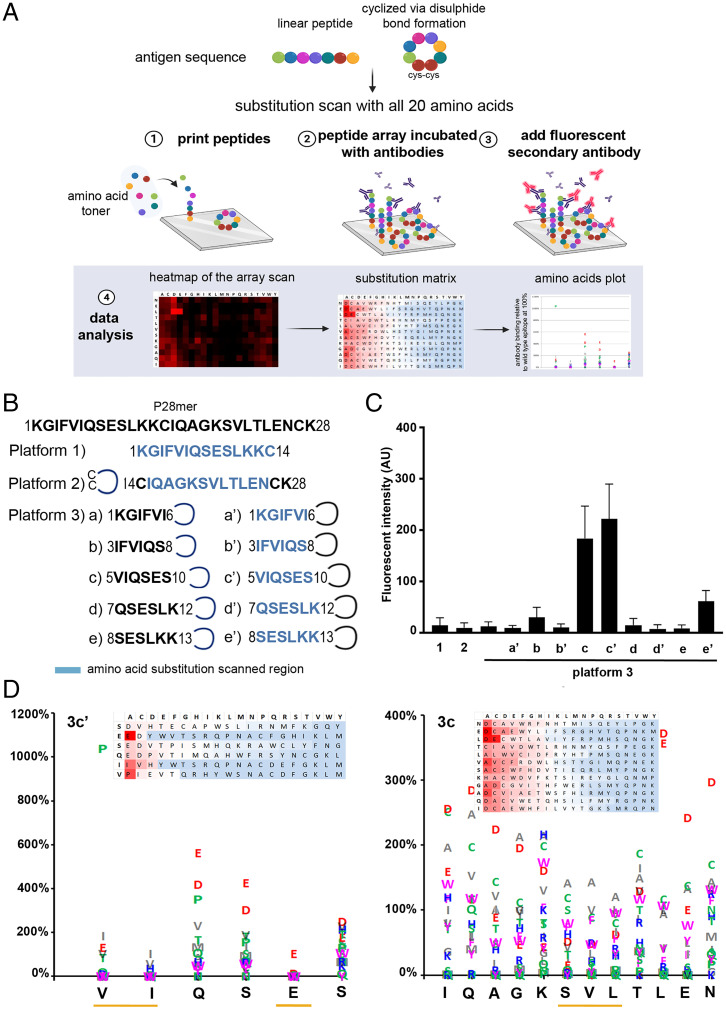
Peptide microarray of the dominant epitope reveals two linear short sequences with key amino acid residues important for PLA2R autoantibody binding. (*A*) Peptide microarray theory and epitope substitution scans. Schematic depiction of the peptide printing process on a microarray of the linear or cyclized antigen sequence (1), antibody incubations (2, 3), followed by binding detection and analysis (4). Surface fluorescent reactivity to an antibody is translated into an amino acid plot and is able to identify conserved residues and variable residues. (*B*) Strategy for peptide sequence optimization. The native P28mer epitope sequence was divided into a linear part, platform 1 (aa1-14); a cyclized part, platform 2 (aa14-28); and a combination of a short 6-amino acids linear region and the cyclized part (platform 3). Blue indicates amino acids involved in the substitution scan. (*C*) Binding detection (fluorescence intensity) of peptides sequences (platforms 1, 2, and 3) to MN patient serum positive for PLA2R autoantibodies. (*D*) Analysis of the epitope substitution scans of peptides platform 3c’ and 3c with substitution matrices and amino acid plot. The amino acid plots showed the effect of any exchange on antibody binding referenced to the wild-type peptide at 100%. Conserved residues are underlined in yellow, indicating the amino acids essential for antibody binding.

**Fig. 2.**
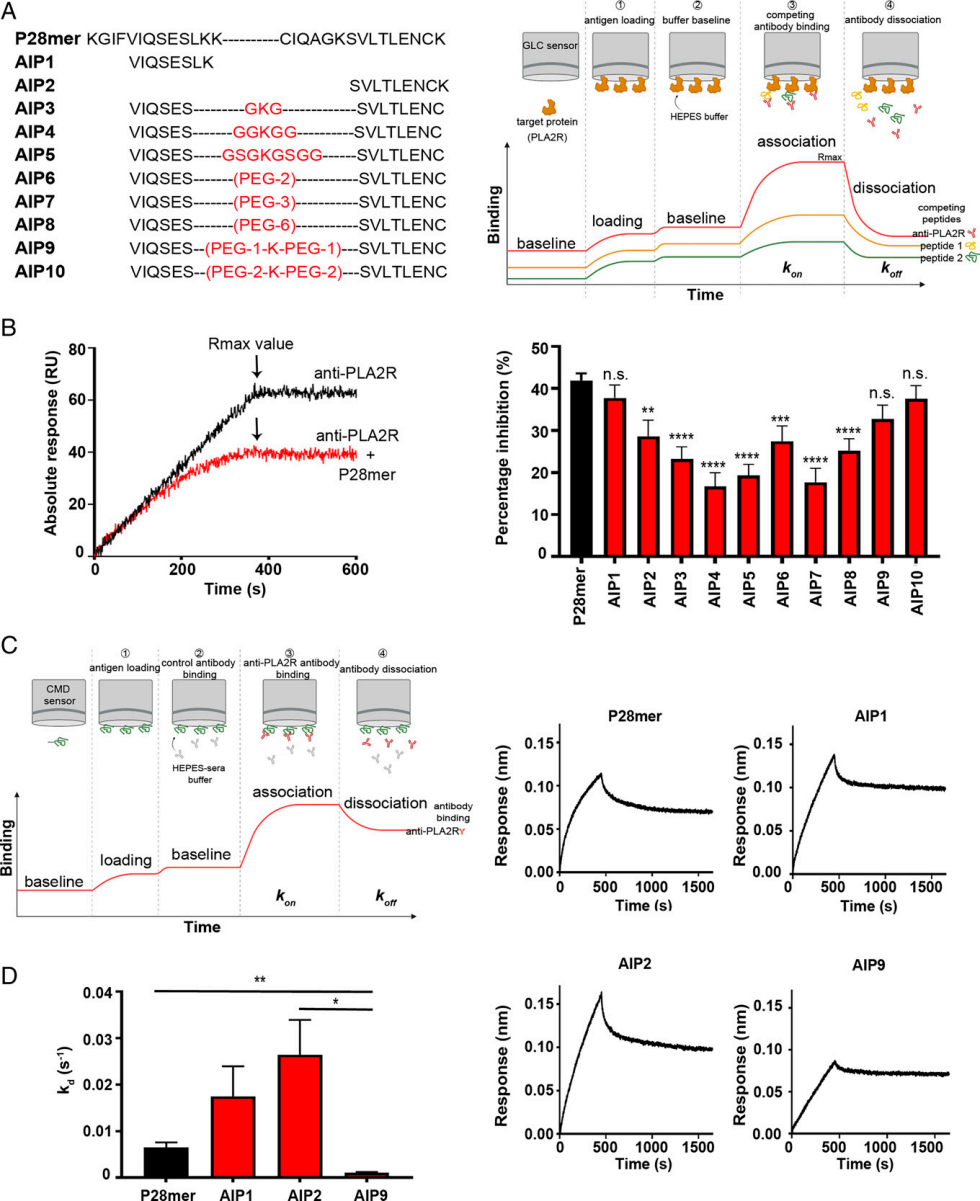
The native epitope folds can be mimicked by inserting linkers between the two important regions. (*A*) List of peptides generated to test potential linkages between the two short linear peptides (AIPs) and schematic of the experimental design for the inhibition assay using SPR. (*B*, *Left*), SPR sensorgram showing real-time binding of MN anti-PLA2R IgG to PLA2R with and without 1 µM P28mer. *Right*, Inhibition analysis of the binding between the human autoantibody and captured PLA2R by the P28mer, and AIPs 1–10 (*n* = 3). (*C*, *Left*), BLI was used to measure the real-time binding of anti-PLA2R positive sera from 27 MN patients to sensors with immobilized P28mer and AIPs as depicted on the schematic. *Right*, Representative sensorgrams demonstrating interaction of PLA2R autoantibody to P28mer, AIP 1, AIP 2, and AIP 9. (*D*) Hill-1 plot analysis of the dissociation phase collected for each response, and dissociation rate calculated (kd) as the 1/k value (*n* = 3). **P < 0.01, ***P < 0.001, ****P < 0.0002; n.s., nonsignificant.

To probe the importance of the amino acids within the PLA2R epitope in human autoantibody recognition, we utilized a printed peptide microarray. This peptide laser printing technology allows the synthesis of peptides (between 15 and 20 amino acids in length) directly onto glass slides followed by single substitution scans, which involves the stepwise substitution of all amino acid positions of a given epitope with all 20 main amino acids (Fig. 1*A*). We first synthesized a stepped series of truncated peptides across the originally discovered 31mer epitope (at both *N*- and C-terminus end) in order to identify the minimum sequence retaining high-affinity binding. The epitope peptide contained three N-terminal amino acids that had no effect on antibody binding, which were subsequently removed, resulting in a 28-amino acid epitope referred to as P28mer (^1^KGIFVIQSESLKKCIQAGKSVLTLENCK^28^) (*SI Appendix*, Fig. S1). We have previously shown that the epitope peptide is a disulphide-bonded structure, and its reactivity to human anti-PLA2R is sensitive to reduction (8). Therefore, the P28mer peptide sequence was divided into a linear region ^1^KGIFVIQSESLKKC^14^ (platform 1) and a cyclic region ^14^CIQAGKSVLTLENCK^28^ (platform 2), where the latter was cyclized by disulphide formation between the *N*- and C-terminal cysteine side chains (Fig. 1*B*). The substitution scan of these two regions was performed by exchange of the native amino acids with all 20 main amino acids. These first scans showed no significant binding of the autoantibody, suggesting that individually, the *N*- and C-terminal regions of the epitope are insufficient (Fig. 1*C*). To further investigate this, a second array (platform 3) combined both parts of the N-terminal region and the cyclic C-terminal region. The N-terminal sequences consisted of five linear peptides with six amino acids each, labeled a) KGIFVI, b) IFVIQS, c) VIQSES, d) QSESLK, and e) SESLKK, with an overlap of two residues across the linear region of the P28mer epitope (amino acids 1–13 KGIFVIQSESLKK), and this was joined to the cyclic region (amino acids 14–27 CIQAGKSVLTLENC). Amino acid substitution scans were performed in the linear (platform 3 a’-e’) or cyclic (3 a-e) region with only Cys14 and Cys27 invariant due to their importance in maintaining the conformation of the epitope (Fig. 1 *B* and *C*). This showed that sequence VIQSES (3 c-c’) was the most antigenic, with little or no reactivity of the autoantibody to the other peptides. Further evaluation of conserved residues showed that amino acid substitution at positions V5, I6, and E9 demonstrated a significant loss of binding compared to the wild-type sequence (Fig. 1*D*). Due to low signal-to-noise ratios, the substitution scan of the C-terminal cyclic stretch IQAGKSVLTLEN resulted in a noisy response pattern, but amino acid positions S20, V21, and L22 hinted at a specific contribution to antibody binding. However, when the SVLTLENCK sequence (AIP2) was tested in solution, the signal improved and suggested an important role in the autoantibody recognition (Fig. 2*B* and *SI Appendix*, Fig. S3*A*). Taken together, these results highlight that the N-terminal VIQSES and C-terminal SVLTLEN regions of the epitope have low tolerance to variation in the sequence compared with the central amino acids, and the conserved residues V5, I6, and E9 appear to be vital for PLA2R autoantibody binding.

To determine the contribution of the conformation of the epitope sequence, the binding of the autoantibody was tested against Endo180 protein, another member of the mannose receptor family with high structural homology of the PLA2R CysR domain (*SI Appendix*, Fig. S2). No interaction was detected, indicating that the autoantibody recognizes specific antigenic amino acids within PLA2R sequence rather than the overall conformation alone.

### The native epitope fold can be mimicked by inserting linkers between the *N*- and C-terminal active peptides.

The peptide microarray analyses implicated the regions ^5^VIQSES^10^ and ^20^SVLTLENCK^28^ within the P28mer epitope in PLA2R autoantibody binding. We next tested whether the two *N*- and C- terminal sequences could be separated by nonnatural sequences and still maintain antigenic reactivity. To that effect, a series of autoinhibitory peptides (AIPs) were designed with various linker regions between ^5^VIQSES^10^ and ^20^SVLTLENC^27^ sequences. The linkers were of varying length and composed of glycine or PEG (polyethylene glycol) spacer elements. AIP 1 and 2 are the two *N*- and C-terminal peptides with no linkers, and AIP 3–10 are peptides with linkers of varying length and type. These peptides were used to inhibit the binding between immobilized PLA2R and human anti-PLA2R antibody by surface plasmon resonance (SPR) (Fig. 2*A*) and competitive ELISA (*SI Appendix*, Fig. S3*A*). The N-terminal peptide VIQSESLK (AIP1) was efficient at blocking antibody binding, giving a similar percentage inhibition (∼40%) compared to the P28mer (Fig. 2*B*). The addition of nonnatural linkers composed of glycine and lysine residues or PEG (AIP3-8) could not recapitulate the native spacing between the two peptides, resulting in an inefficient inhibition (∼20%). However, the PEG-Lys-PEG linkers (AIP9 and 10) did emulate the correct spacing, achieving an inhibition closed to AIP1 and the P28mer. To further characterize the role for these linkers in presenting the peptides more efficiently, we determined the binding affinity of immobilized AIP1, 2, and 9 to 27 MN anti-PLA2R positive sera (Fig. 2*C*). The two short *N*- and C-terminal peptides, AIP1 and 2, had lower affinity than the P28mer. Interestingly, the binding of the PEG-Lys peptide (AIP9) to the antibody was more efficient than the original P28mer peptide as shown using biolayer interferometry (BLI) (Fig. 2*D* and *SI Appendix*, Table S1) and pull-down assay (*SI Appendix*, Fig. S3*B*). These results show that the conformation of the *N*- and C-terminal peptides is crucial for efficient antibody recognition, and the PEG-Lys linker aids the presentation of the antigenic part of the peptides to the autoantibody.

### High-resolution cryoEM structure of human PLA2R reveals a different domain arrangement.

The next step was to map the epitope sequence onto the 3D structure of PLA2R. Current structural models of PLA2R suggest the surface availability of the dominant epitope is pH dependent and inaccessible in a closed conformation (27). However, PLA2R autoantibody binding to its receptor is not pH dependent (8). Therefore, further detailed structural analysis on the PLA2R protein is required to reconcile these discrepancies. We expressed and purified the soluble ECD of PLA2R (residues 21–1397, spanning the N-terminal CysR to the C-terminal CTLD8 region) and performed small-angle X-ray scattering (SAXS) and cryoEM structural analysis.

As previously reported, PLA2R undergoes structural rearrangement at acidic pH (8, 27). Size-exclusion chromatography (SEC) coupled to SAXS analysis was used to quantify these changes. From the SEC elution profile, an increased retention volume at pH 7.2 compared to pH 6.2 supports a conformational change of PLA2R (*SI Appendix*, Fig. S4). The scattering curves and paired-distance distribution functions (P[R]) indicated that PLA2R was elongated and flexible at neutral pH but became compact and rigid at more acidic pH (*SI Appendix*, Fig. S5). For instance, the radius of gyration (Rg) of PLA2R decreased from 60.3 Å to 42.32 Å when the pH was lowered, concomitant with a decrease in maximum particle size (Dmax) from 260 Å to 158 Å (*SI Appendix*, Table S2). The reduction observed in Rg and Dmax is corroborated by analysis of the normalized Kratky plot, where at a neutral pH, PLA2R is less globular than at an acidic pH (*SI Appendix*, Fig. S5).

**Fig. 3.**
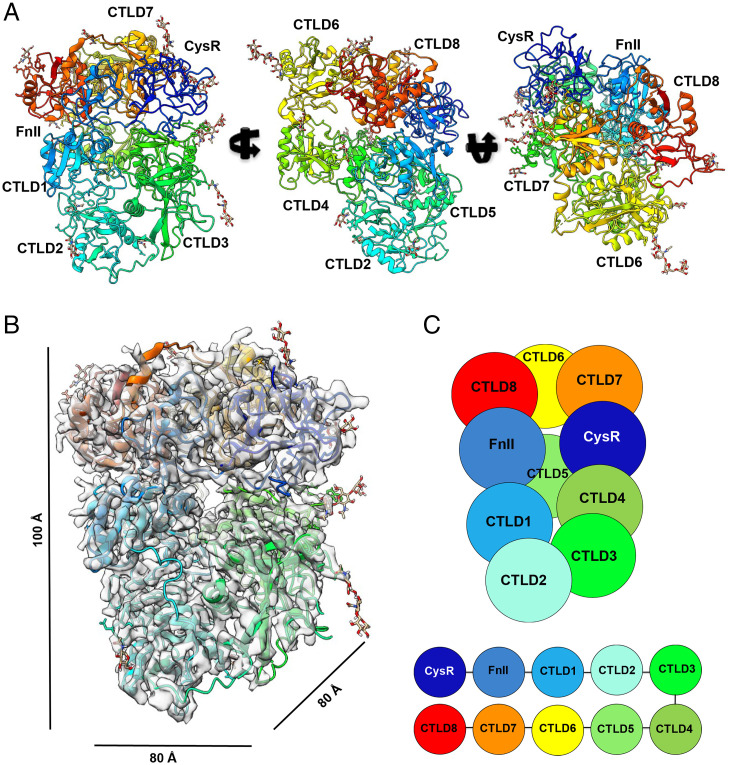
High-resolution cryoEM structure of human PLA2R reveals a different overall domains arrangement. (*A*) Overview of the cryoEM structure of PLA2R, colored by domain subunits and shown in three orthogonal orientations, identifying the domain orientation and glycosylation positions. Resolved glycans are modeled in stick structure (gold). (*B*) View of the refined PLA2R structure in the EM density map highlighting the position of the CysR domain (dark blue, top right). (*C*) Corresponding schematic model of PLA2R showing the colored domains and their arrangement.

For single-particle cryoEM data collection, PLA2R was buffer exchanged to pH 6.2, consistent with the more compact conformation (*SI Appendix*, Fig. S6). The structure of the PLA2R ECD was determined to a resolution of 3.4 Å (*SI Appendix*, Table S3). The cryoEM reconstruction allowed unambiguous arrangement of the individual PLA2R domains throughout the reconstructed map (Fig. 3*A* and *SI Appendix*, Fig. S7). The PLA2R ECD domains pack tightly together, presenting a globular structure with dimensions of ∼80 × 80 × 100 Å (Fig. 3*B*). Each of the tightly packed PLA2R ECDs could be built from the N-terminal CysR to CTLD8 using mostly contiguous density (Fig. 3*C*). It was also possible to build the initial few sugar moieties of the *N*-linked glycans into the cryoEM density, highlighting the dense glycosylation of PLA2R.

The domain arrangements, and therefore the interdomain interactions, are different from those previously reported (27, 28). The N-terminal CysR domain interacts with both CTLD4 and CTLD7. FnII interacts with CTLD8 and CTLD6, and appears to have a substantial loop interaction with CTLD8, although these domain interactions are observed in the closed conformation and could differ at neutral pH.

### Structural insights into the presentation of PLA2R epitope(s).

**Fig. 4.**
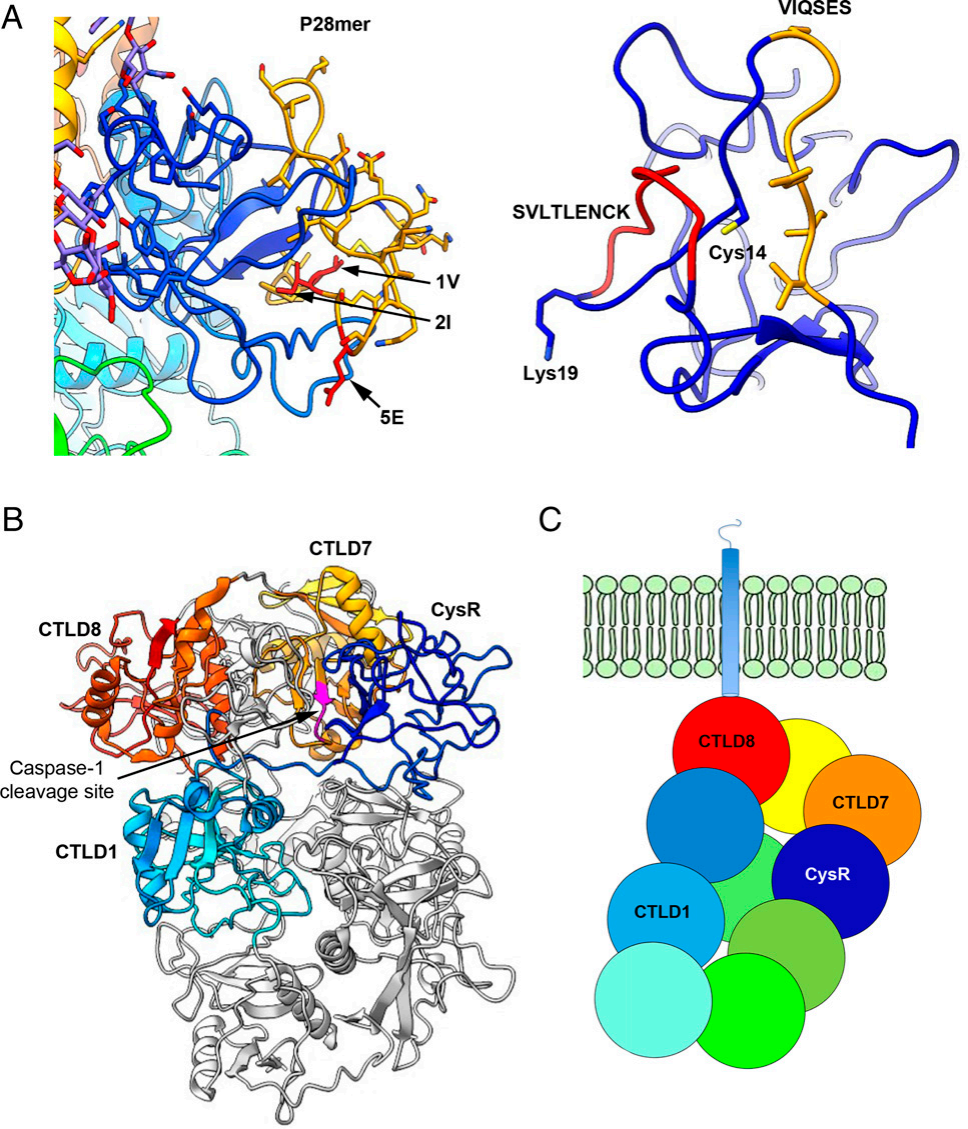
Structural insights into the presentation of PLA2R epitope(s). (*A*, *Left*), Epitope mapping focusing on the CysR domain (blue), highlighting the P28mer (orange), and the key amino acids V, I, and E (red). *Right*, View of the epitope showing the binding cleft and the two antigenic peptides (VIQSES [orange] and SVLTLENCK [red]). (*B*) Structure highlighting domains known to have antibody epitopes along with a cryptic cleavage site for caspase 1 (magenta). (*C*) Model representing the tethering of PLA2R to the cell membrane via the CTLD8 domain and highlighting a hot spot for epitopes localized toward the cell surface.

To understand the properties of the dominant epitope of PLA2R, it is important to determine not only the tertiary structure of PLA2R for the surface availability of the dominant epitope but also its secondary structure within the CysR domain. The density of the CysR domain was complete enough to unambiguously assign residue orientations. The key hydrophobic residues V and I within the VIQSES sequence are located within an accessible cleft on the CysR domain, while the SVLTLENC sequence is entirely surface exposed. The midportion of the P28mer, which does not convey any antibody binding, is not accessible (Fig. 4*A*). Additional antibody epitopes have been identified as being in the CTLD1, 7, and 8 domains of the PLA2R protein (Fig. 4*B*). The domains containing the different epitopes are in close proximity to each other and are localized near the cell surface, as shown on the model (Fig. 4*C*). This cartoon represents the tethering of PLA2R to the cell membrane via CTLD8 and shows how the epitopes may be occluded.

This high-resolution cryoEM structure of PLA2R allowed the accurate mapping of the dominant epitope and its antigenic sequence, and revealed a hot spot of the additional epitopes.

## Discussion

In this study, we discovered the 1) key nonredundant amino acids in the dominant epitope of PLA2R, 2) two essential regions for autoantibody binding, and 3) mapping of the epitope(s) on the high-resolution structure of PLA2R. These key findings improve our understanding of PLA2R immunoreactivity and can now inform the development of antibody inhibitory treatment for MN.

We identified two regions of the dominant epitope (P28mer sequence) by peptide microarray as important for PLA2R autoantibody binding, specifically, the VIQSES and SVLTLENC regions. An in-depth analysis of the epitope substitution scans of both peptides highlighted the importance of the conserved amino acid positions ^1^V, ^2^I, and ^5^E in the N-terminal linear stretch VIQSES, as well as amino acids position ^13^S, ^14^V, and ^15^L in the C-terminal stretch SVLTLENC. We found these two linear peptides could inhibit PLA2R autoantibody binding to PLA2R in solution at similar levels to the P28mer epitope sequence. In addition, linkage of these two regions with a PEG spacer improved the level of reactivity observed with the native P28mer epitope peptide. Interestingly, the intramolecular disulphide bond, which is common to PLA2R, MR, Endo180, and DEC205, family (*SI Appendix*, Fig. S2) is not essential for binding but probably controls the three-dimensional spacing of VIQSES and SVLTLENC.

Although PLA2R exhibits a pH-dependent conformational change, PLA2R autoantibody binding is not pH dependent (8). Indeed, our structure explains why autoantibody binding to the PLA2R epitope is not pH dependent. Of the two constituents that comprise the epitope, VIQSES occupies a surface-accessible cleft, and SVLTLENC is entirely surface exposed on the CysR domain unaffected by compaction of the structure at lower pH. However, enzyme degradation of PLA2R during antigen processing in immune cells may be influenced by structure compaction occurring in intracellular compartments at pH 6.2 or lower to favor or protect parts of PLA2R from immune processing.

The ability of this synthetic AIP9 to interact with higher affinity than the P28mer to PLA2R autoantibodies potentially offers exciting prospects for a more targeted treatment for MN patients. Moreover, the relatively simple structure comprising two short peptides with no disulphide bonds is potentially more stable than the native P28mer epitope peptide, allowing for greater diagnostic and therapeutic potential. This would make it easier and more cost-effective to manufacture on a commercial scale, thereby increasing the potential utility of AIP9 as a future therapeutic. Such a therapeutic might constitute the PLA2R epitope conjugated to the human polymeric immunoglobulin G (IgG) Fc receptor (29) to selectively deplete circulating anti-PLA2R antibodies, as has been demonstrated in an experimental model for selective depletion of anti-HER2 (human epidermal growth factor receptor 2) antibodies using an HER2-Fc receptor construct (30). The possibility now exists to create and test a CysR epitope-FcR construct for ability to provide specific degradation of anti-PLA2R autoantibody in MN patients.

In defining the high-resolution structure for PLA2R, we have unambiguously determined its domain arrangement, which differs from previously reported models (8, 27). The differences in the configuration of the chain of the CTLD domains and overall domain orientations can be attributed to the challenges associated with model building to lower resolution maps. In addition, we have demonstrated the surface availability of the CysR epitope, allowing interaction with anti-PLA2R. Other epitopes in PLA2R have been located to CTLD 1, 7, and 8 domains. Interestingly, the arrangement of the domains CysR, CTLD1, CTLD7, and CTLD8 in this structure identifies a discrete immunogenic patch on PLA2R that induces autoantibody formation and is the target for autoantibody binding. The structure also highlights the accessibility of the sole caspase-1 cleavage site in the CTLD7 domain. Cleavage of cell surface receptors by caspase-1 is common, which raises its possible involvement in the initiation of autoimmunity to PLA2R causing exposure of immunogenic regions, such as the CysR epitope, to antigen processing by the immune system.

In addition to increasing our fundamental knowledge of the availability and surface presentation of epitopes for autoantibodies within PLA2R, the high-resolution structure herein can be applied to the in silico selection of compounds and the development of novel therapeutics.

## Materials and Methods

### Ethical approval of patient samples.

All human samples (serum and antibody eluates) used in this study had been anonymized and were provided by individuals who had given informed consent for research into MN under ethics authority granted by Manchester University NHS Foundation Trust, REC 12/SW/0289 and REC 16/NW/0119. Patient demographic data are in Table 1.

**Table 1.** Patient demographics

Patient characteristics (*n* = 27)	Value
Sex, No. (%)	
Male	20 (74)
Female	7 (26)
Years of age at time of diagnosis, mean (SD)	63 (16)
Ethnicity, No. (%)	
Caucasian	18 (67)
Asian	9 (33)
Anti-PLA2R levels (AU)	>3000

### Peptide microarray.

Peptide microarrays were generated and performed by PEPperPRINT (Heidelberg, Germany) (31, 32). Printed variants of the PLA2R epitope peptides (P28mer) of a maximum of 20 amino acids in length were evaluated. Peptide microarray contained 2214 different peptides printed at least in duplicate and were framed by additional HA control peptide (YPYDVPDYAG) to show the expected spot pattern. Details of the peptides scans are described in Fig. 1*B*. The PEPperCHIP peptide microarrays were preblocked and stained for nonspecific binding of secondary antibody goat anti-human IgG conjugated to DyLight 680 (1:5000) and a control antibody (anti-hemagglutinin) conjugated to DyLigh800 (1:2000) in incubation buffer. The peptide microarray was then incubated with PLA2R autoantibody from one MN patient serum positive for PLA2R at a dilution of 1:100. After washing, the microarrays were stained with the secondary antibody, used in the prestaining, followed by further washing. The autoantibody bound peptides were visualized by LI-COR Odyssey Imaging System. Peptide spot intensity quantification and peptide annotation were executed with PepSlide Analyzer. Data were converted to an amino acid plot for substitution scans conducted on and presented as a percentage binding compared to wild-type peptide sequence.

### Conservation mapping.

The P28mer sequence from human PLA2R protein and the corresponding sequences from the mannose receptor proteins family were acquired from UniProt and submitted to the web-based sequence logo generation tool to analyze the level of amino acid conservation (33, 34).

### Peptides.

Peptides were obtained from ProImmune Ltd. with a purity of at least 80%. AIPs were initially reconstituted in Milli-Q water in aliquots of 0.5 mg, and the peptide concentration was calculated by measuring the absorbance using a spectrophotometer (DeNovix DS-11 FX+, Cambridge Bioscience) at 205 nm. Once peptide concentrations were known, the freshly reconstituted peptides were further diluted into appropriate buffers for each experimental assay.

### PLA2R proteins

Recombinant human PLA2R proteins (full-length ECDs, PLA2R ECD, and the N-terminal half of PLA2R, PLA2R-NC3) were expressed and purified from conditioned cell culture media as described previously (8).

### Binding studies.

#### 1. Inhibition assay.

Real-time binding analysis was evaluated using an SPR instrument (ProteOn, BIO-RAD). ProteOn GLC sensor lanes were preactivated by injection of 18.75 mg/mL EDC (1-ethyl-3-(3-dimethylaminopropyl) carbodiimide) and 1.4 mg/mL NHS (*N*-hydroxysuccinimide, Sigma-Aldrich). Once activated, 20 μg/mL PLA2R-NC3 in 10 mM sodium acetate buffer (pH 4.5) (BIO-RAD) was injected onto sensor chip lanes for immobilization. Ethanolamine was injected to deactivate the sensors from further immobilization.

SPR inhibition assay was achieved with immobilized PLA2R-NC3 and anti-PLA2R IgG with and without AIPs (Fig. 2*B*). Approximately 50% inhibition was exhibited with 1 μM of P28mer peptide. Subsequently, all AIPs were tested in triplicate using the same concentration. First, anti-PLA2R IgG (1/100) in Hepes buffer (10 mM Hepes, 150 mM NaCl (sodium chloride), 0.05% Tween, pH 7.2) with or without 1 μM peptides (AIPs and P28mer) were injected over immobilized PLA2R-NC3 for 300 s at a flow rate of 50 μL/min. Hepes buffer was subsequently flown over, and the dissociation phase was measured for 400 s. The sensors were then regenerated with two successive injections (40 s) of 10 mM NaOH (sodium hydroxide). Each measured response of anti-PLA2R IgG with or without peptides to immobilized PLA2R-NC3 protein was calibrated against nonspecific binding of the sensor to Hepes buffer and Hepes buffer containing antibodies with or without peptides to a blank immobilized sensor. The maximal response was measured for all conditions of anti-PLA2R IgG and percentage inhibition of PLA2R autoantibody binding to PLA2R by AIPs calculated.

#### 2. Direct interaction assay (peptides-antibody).

BLI experiments were conducted using an Octet Red (ForteBio). Octet CDL sensors (coated with a thin layer of carboxymethyl dextran) were activated by EDC/NHS, at the same concentration used for SPR, for 300 s with 1000 rpm agitation. Two hundred micrograms per milliliter of peptides (AIPs and P28mer) in 10 mM sodium acetate buffer pH 4.5 were immobilized onto individual sensors for 600 s. Following immobilization, sensors were deactivated in 1 M ethanolamine for 300 s to prevent further immobilization of proteins to the samples sensors.

The binding interaction between immobilized peptides (P28mer, AIP1, AIP2, and AIP9) and 27 MN anti-PLA2R positive sera (1/200 in Hepes-sera buffer) was assessed. The association phase was measured for 450 s with 1000 rpm agitation. The dissociation phase for each sensor was subsequently measured by incubating the sensors into Hepes-sera buffer for 1200 s at 1000 rpm. Regeneration of the sensor was achieved by six successive washes in 10 mM NaOH for 15 s followed by 15 s in Milli-Q water. The immobilized sensor responses to anti-PLA2R IgG were calibrated against nonspecific binding of each immobilized sensor to Hepes-sera buffer.

The dissociation phase of each response was collected and plotted, and the dissociation rate was calculated (kd) as the 1/k value produced from a Hill-1 plot analysis. The average dissociation rate was calculated for each peptide sensor binding to the 27 MN patients’ sera. Dissociation rates with an R^2^ value <0.9 or χ^2^ value >3 were excluded from the data due to poor fitting.

### PLA2R structure analysis.

#### 1. Small-angle X-ray scattering.

One hundred microliters of purified PLA2R ECD was prepared in a pH 7.2 bis-Tris buffer at 5 mg/mL. PLA2R was measured using SEC-SAXS on beamline B21 at Diamond Light Source (DLS). Forty-five microliters of PLA2R was loaded onto a Superdex 200 3.2/300 increase column (GE Healthcare) equilibrated with bis-Tris at either pH 7.2 or pH 6.2. SEC-SAXS experiments were measured at a flow rate of 0.075 mL/min with 1 s exposures per frame over 620 frames. The data were collected on an Eiger × 4M detector (Dectris) using a beam with a wavelength of 0.95 Å and a sample-to-detector distance of 2.7 m.

Raw SAXS data images were processed using DLS’s DAWN (35) processing pipeline at the beamline to produce normalized, integrated 1-D unsubtracted SAXS curves. SEC-SAXS frame selections and buffer subtractions were performed with CHROMIXS (36). PRIMUS was used for all processing, with the exception of flexibility plots, where ScÅtter was used. Output files were produced for AMBIMETER to estimate structural uniqueness (37).

Comparison of the final structure of PLA2R from the cryoEM structure was computed using CRYSOL (38). Additional glycans were built onto the PLA2R structure using the GlyProt online server (39).

#### 2. Cryogenic transmission electron microscopy.

CryoEM grids were prepared using 0.3 mg/mL PLA2R prepared in bis-Tris pH 6.2 buffer. Three microliters of PLA2R was applied to either C-flat 2/2 on 300 mesh Cu grid with 20 nm carbon film (Protochips) or Quantifoil Cu 300 mesh R2/2 grids (Quantifoil) and plunge-frozen in liquid ethane using a Vitrobot Mark IV (Thermo Fisher). C-flat grids were screened at the cryoEM facility at the University of Leeds. Grids were images on a Titan Krios G3 electron microscope (Thermo Fisher Scientific) equipped with a Falcon III camera (Thermo Fisher Scientific) in linear mode. Quantifoil grids were screened at the University of Manchester on the Polara G2 electron microscope (FEI) equipped with a K2 Summit camera (Gatan) in counting mode.

Data collections for both grids were carried out at Electron Bio-Imaging Centre (eBIC) on Titan Krios IV at 300 kV with a K3 camera (Gatan) in superresolution counting mode, with C-flat grids imaged at 0° (BI22724-15) and Quantifoil grids at 30° (BI22724-12). A Quantum GIF energy filter (Gatan) was used with a slit width of 20 eV to remove inelastically scattered electrons. For both data collections, 40 movie frames were recorded, using a dose rate of 14.8 e^−^/Å^2^/s per frame for a two-second exposure, for a total dose of 42.9 e^−^/Å^2^ at a pixel size of 0.83 Å (superresolution pixel 0.415 Å). A total of 3702 images were collected at 0°, and 2137 images were recorded at 30°. Further details can be found in *SI Appendix*, Table S2.

All images were imported into cryoSPARC (v3.2). Motion correction and Fourier cropped to 0.815 Å/pix and CTF (contrast transfer function) estimation were carried out using patch motion correction and patch CTF estimation. Images taken at 0° were curated, and any images with a defocus lower that −3 µm, a resolution higher than 4 Å, and an ice thickness of greater than 1.1 were removed, resulting in 2015 images (1687 removed). For images taken at 30°, any images with a defocus lower than −3 µm, a resolution higher than 5 Å, and an ice thickness greater than 1.1 were removed, resulting in 1455 images (682 removed).

Each data set was initially processed separately. Particles were picked using a Gaussian blob (80–130 Å) from 100 images of the 0° data set, extracted in a 256 pixel box, binned 4 × 4 (64 pixel box), and subjected to 2D classification. The best templates were used to template pick all images from both data sets. This resulted in 1.7 million and 1.2 million particles from the 0° and 30° data sets, respectively. These particles were extracted in 256-pixel boxes and binned 4 × 4 (64 pixel box) for 2D classification. The good classes were selected, and five ab initio models were generated using 100,000 particles from the 30° particle picks. These initial models were used by both data sets for heterogeneous refinements of particles selected by 2D classification. The particles from the best model, picked by the highest resolution, were used for generating five more initial models, followed by heterogeneous refinement. This was repeated until the resolution of the refinements was consistent across the models. From here, the best particles were re-extracted in 256-pixel boxes and refined by nonuniform refinement. The particles were then used to train a Topaz model (40) for each of the 0° and 30° data. The above processing was repeated until the resolution remained stable. The particles were then combined and refined again with no increase in the resolution. The 30° data reached the highest resolution, so 0° is not reported. The particles were then locally motion corrected, but the resolution decreased.

#### 3. Model building and refinement.

Initial domain placement was based upon the structure of the related protein, DEC205 (PDB 7JPT) (20). The sequence of PLA2R was mapped onto the structure of DEC205 using HHpred (41). This structure was placed into the map using Phenix.dock_in_map (42). Inspection of the map and model in Coot (43) revealed in-domain loops and linkers that were not resolved in the map. These residues were removed, and the model was broken up into rigid groups, corresponding to domains or pairs of domains. Several rounds of refinement using Phenix.real_space_refine (44) with rigid body fitting of domains, model morphing, and simulated annealing, imposing secondary structure restraints, introducing model-based restraints using the crystal structure of seventh CTL domain of human PLA2R (PDB 6JLI) (28), and rebuilding using both Coot and Isolde (45), yielded the final model, which had good geometry and agreement with the map.

The coordinates of the atomic model of PLA2R have been deposited in the Protein Data Bank with accession code 7QSR. The cryoEM map has been deposited into the Electron Microscopy Data Bank with accession code 14077, and the raw data of both the 0° and 30° tilt data are available on EMPIAR with accession code 10915. The SAXS data and associated models are deposited in the Small Angle Scattering Biological Data Bank, with accession codes SASDNP3 (pH7.2) and SASDBQ3 (pH6.2).

### Statistical analysis.

Prism 8 software was used to present data collected graphically, and statistical significance was calculated using a two-tailed *t* test or two-way analysis of variance (GraphPad). Error bars display the SEM. Statistically significant trends were determined as a *P* value of <0.05 (*). **P < 0.01, ***P < 0.001, ****P < 0.0002.

## Supplementary Material

Supplementary File

## Data Availability

Structure and EM map data have been deposited in the Protein Data Bank (7QSR) (46) and the Electron Microscopy Data Bank (EMD-14077) (47).
