# Supplementary material for: Structure of PLA2R reveals presentation of the dominant membranous nephropathy epitope and an immunogenic patch
Source: Proc Natl Acad Sci U S A. 2022 Jul 11;119(29):e2202209119. doi: 10.1073/pnas.2202209119 (PMC9303975; doi:10.1073/pnas.2202209119)
Supplement: Supplementary File [file pnas.2202209119.sapp.pdf]

PNAS

Supplementary Information for
Structure of PLA2R reveals presentation of the dominant membranous nephropathy epitope and an immunogenic patch

*Maryline Fresquet[1,2], *Michael P. Lockhart-Cairns[1,2], *Samuel J. Rhoden[1,2], Thomas A. Jowitt[1], David C. Briggs[3], Clair Baldock[1,2], Paul E. Brenchley[4], Rachel Lennon[1,2,5]

These authors contributed equally to this work.

Corresponding authors:

Maryline Fresquet: Wellcome Centre for Cell-Matrix Research, Michael Smith Building, University of Manchester, M13 9PT, UK.

Email: maryline.fresquet@manchester.ac.uk

Rachel Lennon: Wellcome Centre for Cell-Matrix Research, Michael Smith Building, University of Manchester, M13 9PT, UK.

Email: Rachel.Lennon@manchester.ac.uk

This PDF file includes:

Supplementary text
Figures S1 to S7
Tables S1 to S3

Supplementary Information Text

Supplementary materials and methods

Figure S3A- Competition ELISA
ELISA plates (ThermoScientific, 3455) were coated with PLA2R-NC3 overnight (4°C) at a concentration of 125 ng/mL diluted in 0.1 M sodium bicarbonate buffer (pH 9.5). Next, the ELISA plates were blocked in SuperBlock buffer (ThermoScientific, 37515) at 4°C overnight, stored and used within three days. Two MN patients' sera were diluted 1 in 400 in SuperBlock (0.05% Tween-20 and protease inhibitor cocktail minus EDTA) and incubated with and without 1000 nM peptides (AIP 1-10) and P28mer at 4°C overnight. Following pre-incubation, samples were then added to a PLA2R-NC3 coated ELISA plates at room temperature with constant shaking for two hours. The plates were then excessively washed in PBS-T (> 7 times) before incubation with anti-human IgG-HRP conjugate diluted 1 in 25,000 (Jackson Labaoratory) in SuperBlock buffer (0.05% Tween-20) and added to each well of the ELISA plates. Again plates were incubated for two hours at room

temperature with constant shaking. The solutions were removed and excessively washed in PBS-T. Finally 100 µL of tetramethylebenzadine (Sigma-Aldrich, T4444) was added to the ELISA plates and incubated at room temperature and after 10 minutes, 50 µL of 1 M sulphuric acid was then added to stop the reaction. The absorbance of each well was measured using a plate reader at 450 nm. Healthy human sera only, healthy human sera plus peptides and secondary only controls were also included in each assay to subtract background signals from the measurements acquired. Percentage peptide inhibition of PLA2R autoantibodies binding to PLA2R-NC3 was then calculated.

Figure S3B – Pull down assay

2 mg of MyOne^{TM} carboxylic acid dynabeads (Invitrogen, UK) were activated by incubation in 18.75 mg/mL EDC (N-3-dimethylaminopropyl-N'-ethylcarbodiimide hydrochloride, Sigma-Aldrich) and then covalently immobilised with 800 µg/mL of PLA2R-NC3, peptides (P28mer, scrambled P28mer, AIP 9) in 15 mM MES buffer pH 6 or buffer alone overnight at 4°C. The magnetic beads were precipitated from samples and subsequently deactivated by incubation in 1 M ethanolamine (BIO-RAD) for 1 hour (room temperature and agitation). Ethanolamine was then removed and the magnetic beads were blocked for nonspecific binding by a 1 hour incubation with SuperBlock buffer at room temperature. Two MN patients' sera were diluted to 1:400 in SuperBlock buffer (0.05% Tween-20) and incubated (overnight, 4°C with agitation) with either blank, PLA2R-NC3 or peptides immobilised magnetic beads. Magnetic beads were then precipitated and the supernatant collected from each condition, then the beads were washed thrice in PBS-T. Antibodies bound to the magnetic beads were eluted with acidic elution buffer (0.1 M glycine pH 2.2) and the eluate from each condition were neutralised with 1 M Tris buffer (pH 8). The supernatant and eluate from magnetic beads (Blank, PLA2R-NC3 immobilised, P28mer, scrambled P28mer and AIP 9) were analysed by PLA2R-NC3 coated ELISA plates. Antibodies bound to the immobilised PLA2R-NC3 or tested peptides were evaluated by calculating the percentage of PLA2R autoantibody reactivity to PLA2R-NC3 in the eluate relative to the total antibodies reactive to PLA2R-NC3 in the supernatant detected by ELISA.

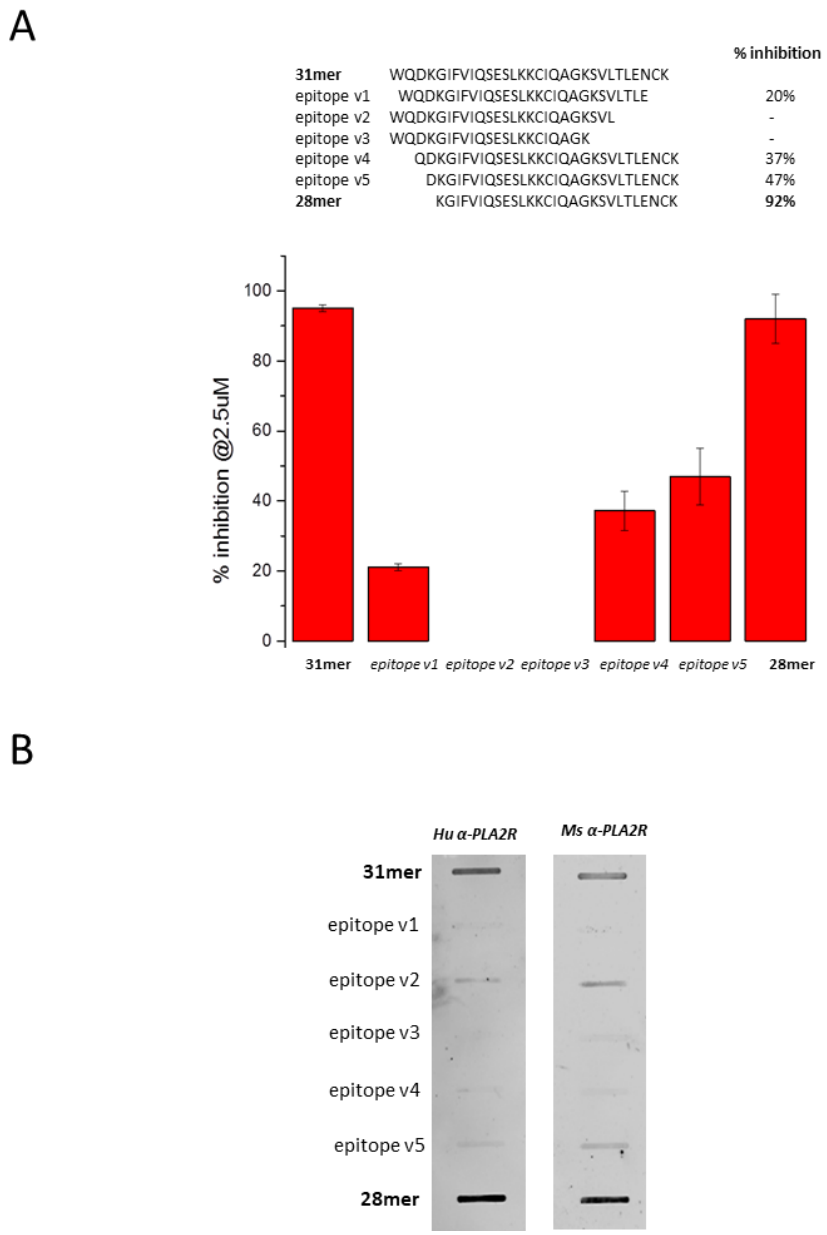

Fig. S1: Determining the smallest reactive epitope peptide.
A) A series of 6 truncated peptides across the 31mer epitope sequence (both N- and C-terminus truncation) were synthesized to identify the minimum sequence retaining high binding activity to anti-PLA2R autoantibody. Truncated peptides (epitope v1-5 and 28mer) were tested by SPR for their potential to inhibit binding of immobilized purified human anti-PLA2R antibody to a PLA2R fragment. At 2.5µM concentration of peptides, the 28mer version reached 92% inhibition, equivalent to the original 31mer. B) Slot blot analysis of 10 µg of 31mer peptide and truncated versions of the epitope sequence using purified human autoantibody (at 1:1000 dilution) and mouse monoclonal antibody to PLA2R (clone 12-5-6 at dilution 1:5000) demonstrating a strong signal (direct binding) to the 28mer peptide. All measures were done in triplicate.

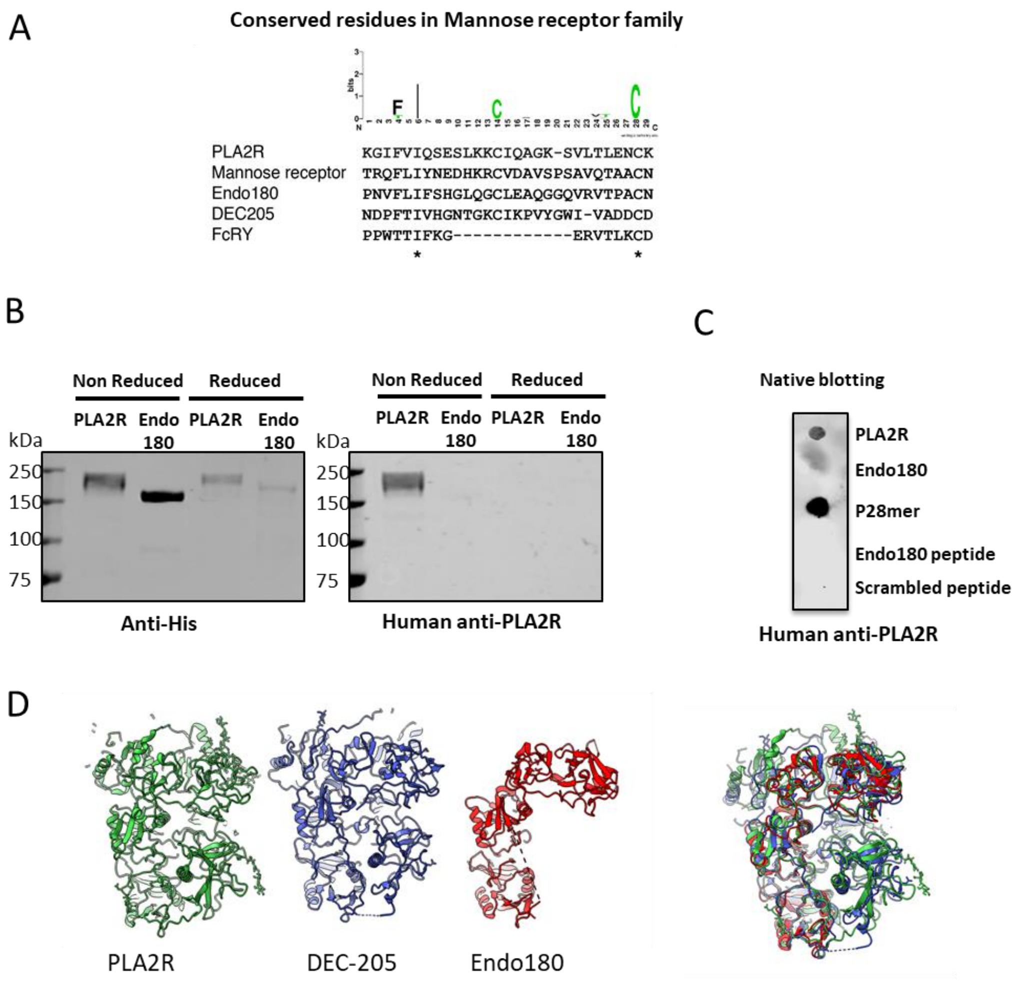

Fig. S2: PLA2R autoantibodies incapable of binding to Endo180 potentially due to lack of surface cavity in the P28mer epitope region.

A) Sequences from the P28mer region in the mannose receptor family proteins were subjected to consensus mapping by weblogo. Residues conserved in all mannose receptor proteins are indicated with *. B) Western blot analysis of PLA2R and Endo180 under non reduced and reduced conditions using anti-His and human PLA2R autoantibody. C) Dot blot analysis of PLA2R and Endo180 proteins, P28mer peptide and the equivalent peptide region in Endo180 against MN PLA2R autoantibody under native condition. D) Comparison of the PLA2R, DEC-205 (5ao5) and Endo-180 (7jpt) structures and their overlays. The RSMD values generated were 3.184 Å RMSD for analysis between PLA2R and Endo180, 3.558 Å RMSD for DEC-205 and Endo180, where Endo180 was used as reference.

A

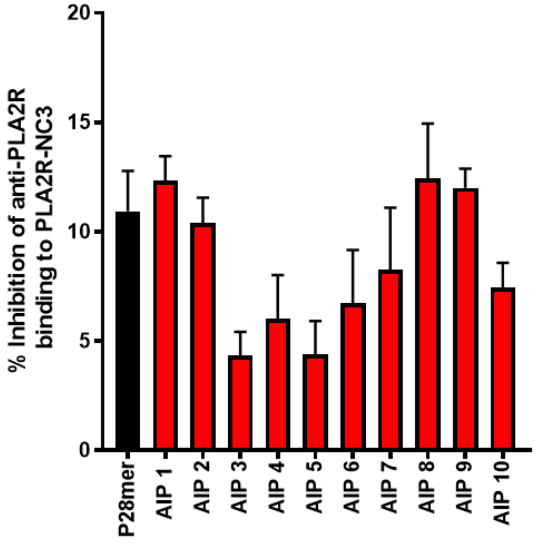

B

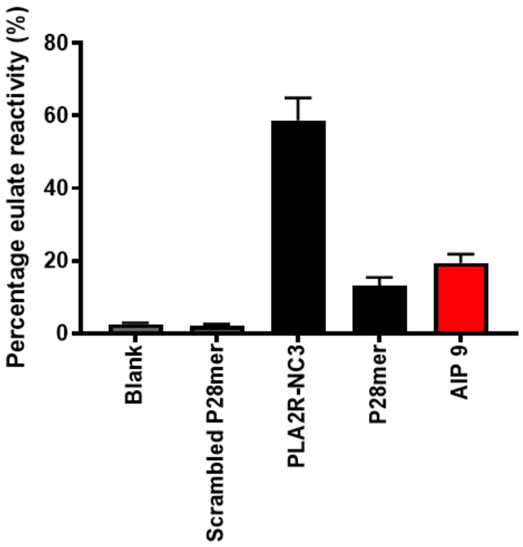

Peptide	% Eluate Response (±SD)
Blank	2.57 (0.83)
Scrambled P28mer	2.17 (0.9)
PLA2R-NC3	58.67 (15.2)
P28mer	13.11 (5.62)
AIP 9	19.44 (5.83)

Fig. S3: Inhibitory effect of AIPs measured by inhibition ELISA and pull down assay.
A) Competitive ELISA of 2 anti-PLA2R positive MN sera incubated with 1µM P28mer or AIP1-10 and binding inhibition measured against PLA2R-NC3 (n=3). B) Pull down assay of PLA2R autoantibodies from 2 patient serum samples using magnetic beads coated with blank, scrambled P28mer, PLA2R-NC3, P28mer and AIP 9. The captured antibodies were subsequently eluted and their reactivity assessed by direct ELISA (binding to the immobilized PLA2R-NC3).

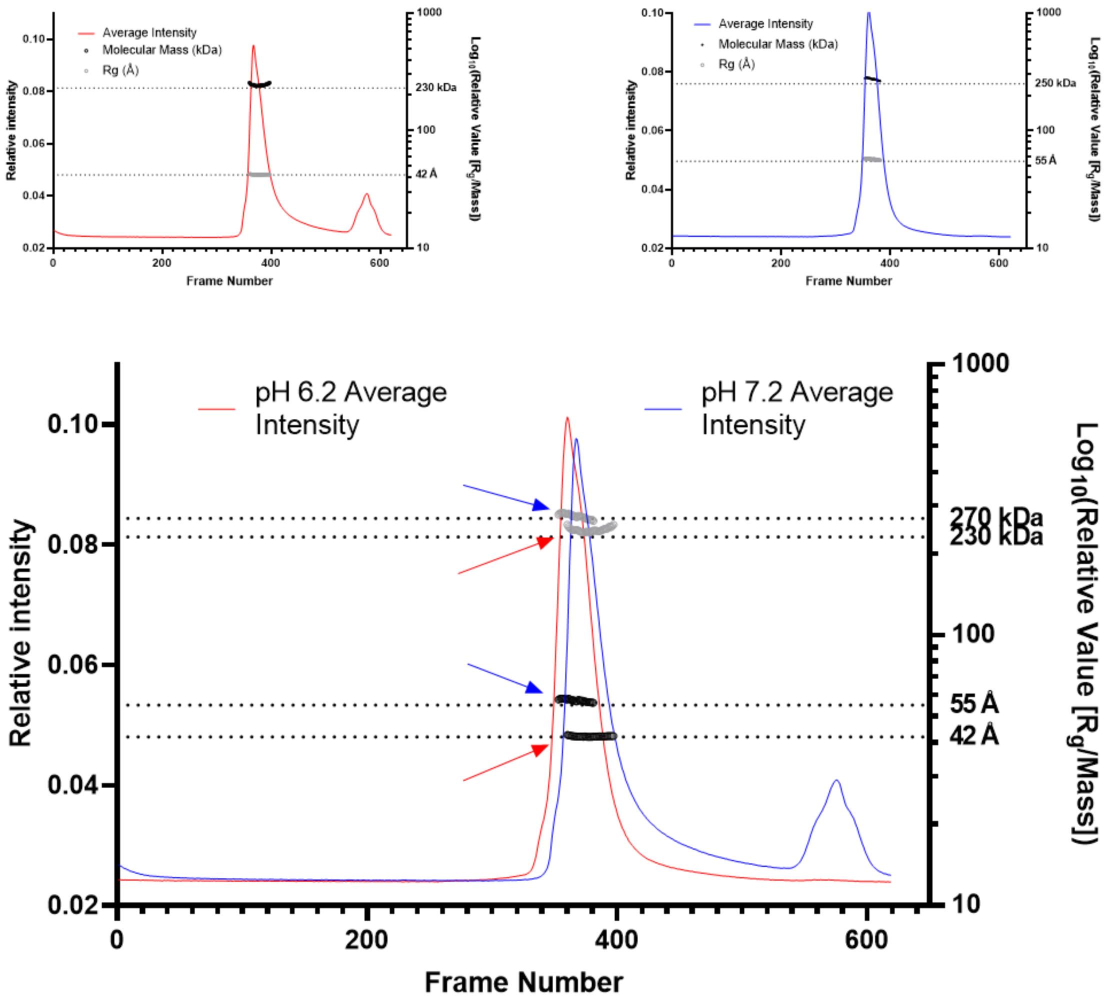

pH	Buffer start	Buffer finish	Total	Sample start	Sample finish	Total
6.2	229	305	76	360	397	37
7.2	262	320	58	353	381	28

Fig. S4: pH-dependent conformational changes
Relative X-ray scattering intensity plots of SEC elution from PLA2R in pH 6.2 (blue) and pH 7.2 (red) buffers. The grey points show the estimates molecular mass per frame of PLA2R at the differing pH and the black points show the differing Rg per frame. The table displays which fractions were taken for the sample and the buffer in each case.

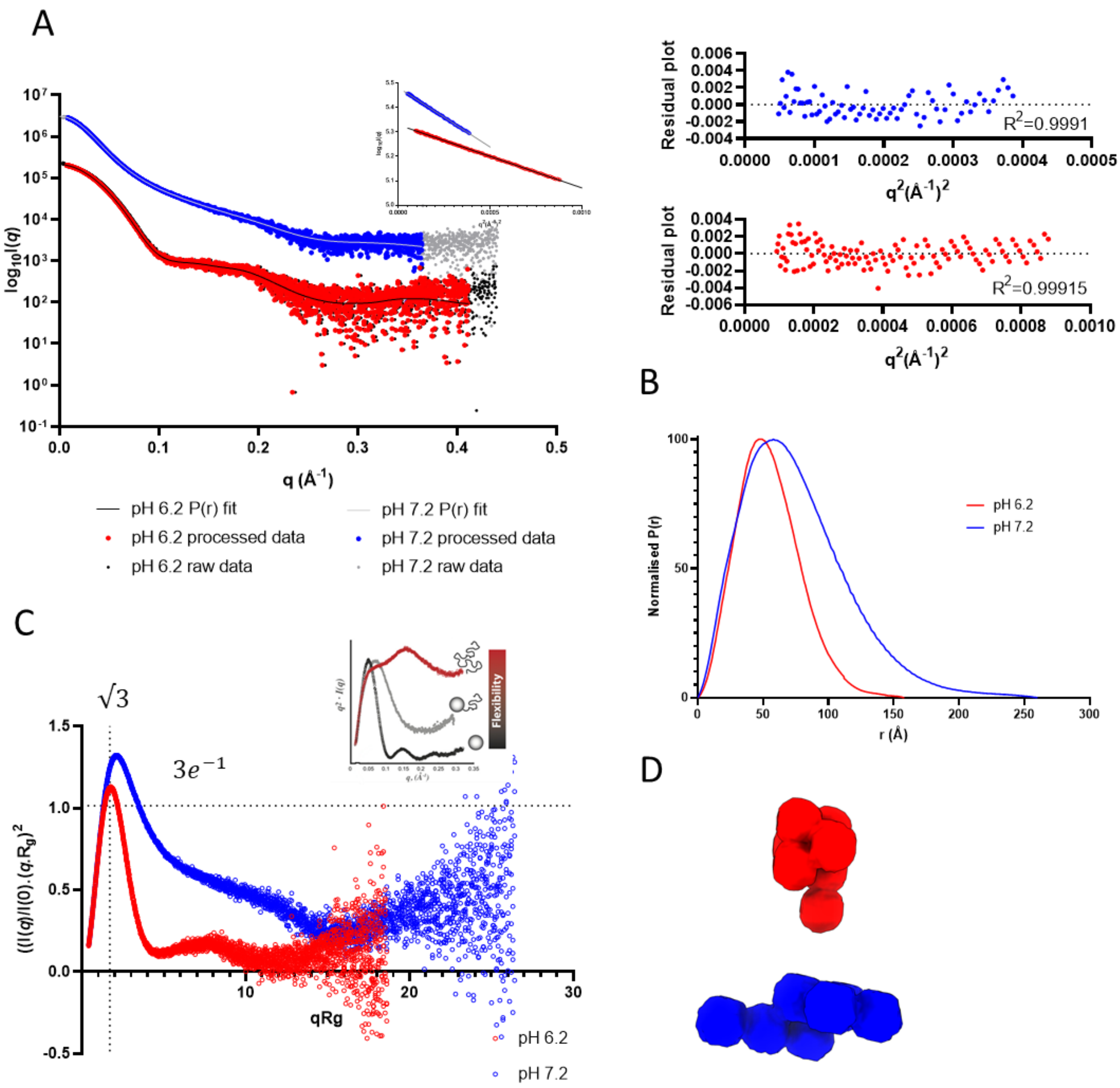

Fig. S5: SAXS data processing at pH 6.2 and 7.2

A) Processed scattering data for PLA2R at pH 6.2 & 7.2 from SEC-SAXS data. Collection parameters can be found in supplemental table 2. The pair distance distribution function (P(r)) was computed in ScÅtter and the inverse Fourier transform is plotted against the scattering data, showing high agreement. The Guinier plot is presented in the inset and the corresponding residuals on the right panel. B) The P(r) of the scattering data in A showing the centre of Shannon bins in coloured circles. C) Normalised Kratky plot showing how the pH of 6.2 provides a more compact arrangement for PLA2R where the peak sits on the crosshairs as expected for a globular protein. PLA2R at pH 7.2 shows a more open and extended conformation where the peak is offset to the right of the crosshairs. D) Most likely conformation predicted by AMBIMETER showing the change in the conformation at pH 6.2 (red) and pH 7.2 (blue). The spheres do not represent domains but rather the shape topology depicted from seven beads.

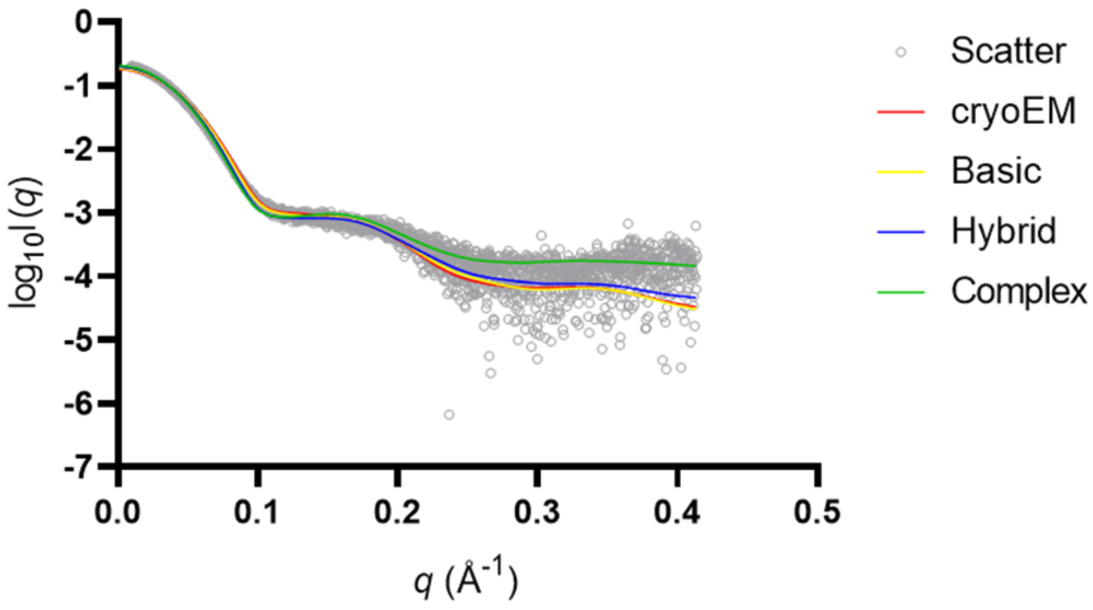

Model	X^2	Rg (Å)
cryoEM	258.81	39.30
Basic	165.33	37.67
Hybrid	42.77	36.93
Complex	11.89	40.69

Fig. S6: SAXS data to model comparison
Models generated from GlyProt with the cryoEM model (red) with basic glycosylation (yellow), hybrid glycosylation (blue) or complex glycosylation (green) were compared to the scattering curve of PLA2R at pH 6.2 using CRYSOL. The table below displays the X^2 values and the calculated Rgs for the models. The experimental Rg is 42.32 Å.

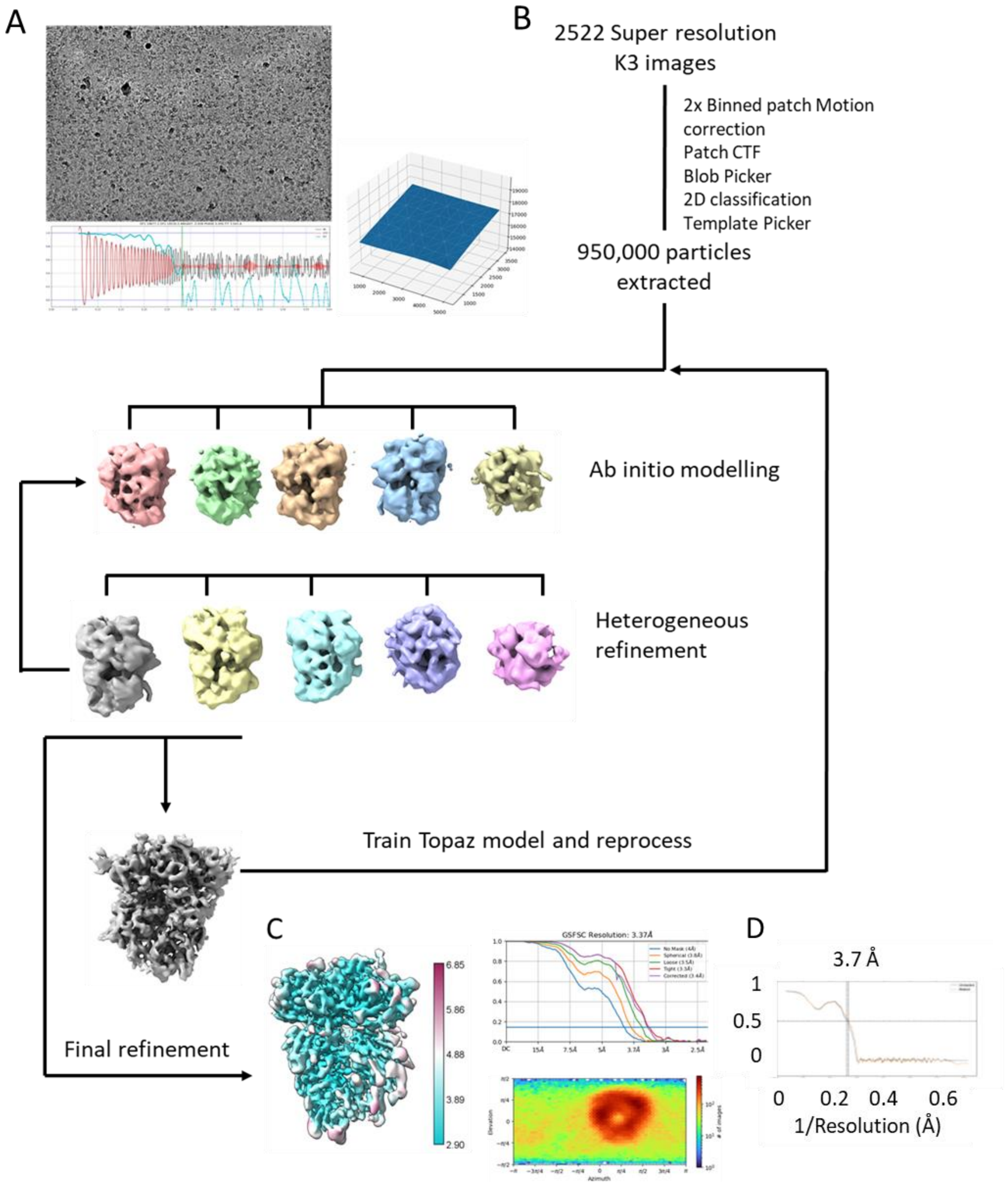

Fig. S7: PLA2R cryoEM workflow
A) An exemplar 30° tilted image with corresponding global and patch CTF estimation. B) Entire pipeline showing the steps from images to local refinement. C) Final refined model of PLA2R with FSC and angular assignment of particles. D) Map to model FSC with cut-off at 0.5, showing 3.7 Å resolution.

Table S1: **Dissociation off-rates (k_d values) for P28mer, AIP1, AIP2 and AIP9 measured by BLI**

The direct interaction of the 4 selected peptides (P28mer, AIP1, AIP2 and AIP9) was tested against 27 MN patient serum samples using BLI and the off-rates determined. The dissociation phase of each response was fitted to Hill-1 plot analysis, and off-rate (k_d) calculated. Dissociation rates with $R^2<0.9$ and $X^2>3$ were excluded due to poor fitting.

PATIENT NUMBER	Peptide offrate k_d (s^{-1})			
	P28MER	AIP1	AIP2	AIP9
1	3.71E-03	1.36E-04	4.51E-03	2.17E-03
2	1.54E-03	9.13E-04	5.31E-03	2.52E-03
3	1.38E-02	2.79E-02	5.65E-03	1.84E-03
4	6.35E-03	1.14E-03	6.49E-02	2.12E-04
5	8.00E-03	5.18E-04	5.31E-03	1.39E-03
6	4.48E-04	2.07E-03	2.49E-03	-
7	8.26E-03	7.23E-02	6.09E-02	1.65E-03
8	6.28E-03	1.94E-02	7.92E-03	-
9	7.38E-03	1.37E-03	-	-
10	7.21E-03	-	-	6.53E-04
11	1.46E-02	-	1.23E-01	5.34E-04
12	6.47E-03	-	6.64E-03	1.84E-03
13	2.70E-04	1.24E-01	-	9.70E-04
14	1.26E-03	1.96E-03	1.71E-03	-
15	6.56E-03	2.93E-03	-	1.52E-03
16	7.37E-03	-	3.53E-03	-
17	2.62E-02	2.77E-02	5.18E-02	8.06E-04
18	9.44E-04	-	5.65E-03	4.45E-04
19	2.31E-04	1.71E-02	6.49E-02	4.94E-04
20	5.23E-03	1.27E-02	2.49E-03	7.77E-05
21	5.68E-03	1.26E-02	1.39E-04	5.70E-04
22	1.69E-02	-	3.04E-02	-
23	9.20E-04	1.08E-03	1.02E-01	1.30E-03
24	1.51E-03	1.01E-03	2.05E-03	2.25E-03
25	4.14E-03	2.40E-02	1.71E-03	4.53E-04
26	7.21E-03	3.18E-03	3.53E-03	6.31E-04
27	6.35E-03	1.32E-02	5.18E-02	5.70E-04
**Average**	**6.47E-03**	**1.75E-02**	**2.64E-02**	**1.09E-03**

Table S2: SAXS parameters

Parameters<br>SASBDB	PH 6.2<br>SASDNQ3	PH 7.2<br>SASDNP3
Low q	0.0096	0.0080
High q	0.4139	0.3641
Mass estimates (kDa)		
Vc	211.97	231.98
MoW	235.94	234.28
Baysian Inference	242.63	242.63
**Reciprocal**		
Rg	42.3	59.97
I(0)	0.2132	0.3051
Guinier points	37 - 149	22 - 91
**Real**		
Rg	42.32	60.25
I(0)	0.2132	0.3052
Dmax	158	260
Estimate	0.7737	0.7027
**Modelling**		
Ambimeter (shape categories/score)	3/0.4771	82/1.914
CRYSOL of cryoEM structure (X^2)	258.81	

Table S3: cryoEM parameters

**PLA2R (EMD-14077) (PDB ID: 7QSR)**	
Data collection and processing	
Magnification	81,000x
Voltage (kV)	300
Electron exposure (e/Å^2)	42.9
Defocus range (μm)	−0.8 to −2.0
Pixel size (Å)	0.815
Symmetry imposed	C1
Initial particle images (no.)	950,000
Final particle images (no.)	221,000
Map resolution (Å)<br>FSC threshold	3.4<br>0.143
Map resolution range (Å)	2.9 to 6.9
Refinement	
Initial model used (PDB code)	7JPT
Model resolution (Å)<br>FSC threshold	3.4<br>0.143
Map sharpening B factor (Å^2)	−132
Map to Models resolution (Å)	3.7
**Model composition**<br>Chains<br>Atoms<br>Residues<br>Water<br>Ligands	 6<br>21649 (Hydrogens: 10459)<br>Protein: 1335 Nucleotide: 0<br>0<br>BMA: 4<br>NAG: 17<br>MAN: 2
Bonds (RMSD)<br>Length (Å) (# > 4σ)<br>Angles (°) (# > 4σ)<br>MolProbity score<br>Clash score	 0.003 (0)<br>0.715 (5)<br>1.97<br>8.79
Ramachandran plot (%)<br>Outliers<br>Allowed<br>Favored<br>Rotamer outliers (%)	 0.30<br>8.04<br>91.66<br>0.50
ADP (B-factors)<br>Iso/Aniso (#)<br>min/max/mean<br>Protein<br>Nucleotide<br>Ligand<br>Water	 11190/0<br><br>105.26/326.00/184.62<br>---<br>156.77/320.57/236.86<br>---
